# Manifold Based Optimization for Single-Cell 3D Genome Reconstruction

**DOI:** 10.1371/journal.pcbi.1004396

**Published:** 2015-08-11

**Authors:** Jonas Paulsen, Odin Gramstad, Philippe Collas

**Affiliations:** Department of Molecular Medicine, Institute of Basic Medical Sciences, Faculty of Medicine, and Norwegian Center for Stem Cell Research, University of Oslo, 0317 Oslo, Norway; Weizmann Institute of Science, ISRAEL

## Abstract

The three-dimensional (3D) structure of the genome is important for orchestration of gene expression and cell differentiation. While mapping genomes in 3D has for a long time been elusive, recent adaptations of high-throughput sequencing to chromosome conformation capture (3C) techniques, allows for genome-wide structural characterization for the first time. However, reconstruction of "consensus" 3D genomes from 3C-based data is a challenging problem, since the data are aggregated over millions of cells. Recent single-cell adaptations to the 3C-technique, however, allow for non-aggregated structural assessment of genome structure, but data suffer from sparse and noisy interaction sampling. We present a manifold based optimization (MBO) approach for the reconstruction of 3D genome structure from chromosomal contact data. We show that MBO is able to reconstruct 3D structures based on the chromosomal contacts, imposing fewer structural violations than comparable methods. Additionally, MBO is suitable for efficient high-throughput reconstruction of large systems, such as entire genomes, allowing for comparative studies of genomic structure across cell-lines and different species.

## Author Summary

Understanding how the genome is folded in three-dimensional (3D) space is crucial for unravelling the complex regulatory mechanisms underlying the differentiation and proliferation of cells. With recent high-throughput adaptations of chromosome conformation capture in techniques such as single-cell Hi-C, it is now possible to probe 3D information of chromosomes genome-wide. Such experiments, however, only provide sparse information about contacts between regions in the genome. We have developed a tool, based on manifold based optimization (MBO), that reconstructs 3D structures from such contact information. We show that MBO allows for reconstruction of 3D genomes more consistent with the original contact map, and with fewer structural violations compared to other, related methods. Since MBO is also computationally fast, it can be used for high-throughput and large-scale 3D reconstruction of entire genomes.

## Introduction

Understanding genomes in three dimensions (3D) is a fundamental problem in biology. Recently, the combination of chromosome conformation capture (3C) methods with next-generation sequencing, such as 5C [1], Hi-C [2], TCC [3], and GCC [4], has enabled the study of contact frequencies across large genomic regions or entire genomes. These methods consist in crosslinking a large sample of cells followed by restriction enzyme digestion and ligation. Ligated DNA molecules are isolated, and sequenced using massively parallel paired-end sequencing. The end-result is typically a large matrix containing interaction (ligation) frequencies between all regions of the genome under study in the cell population. While such matrices can be visualized and analyzed directly [2], determining the 3D structure corresponding to the interaction frequency matrix has been of steady increasing interest in the fields of computational biology and genomics. However, such 3D genome reconstruction is challenging due to the sparse and noisy nature of the data, the fact that the matrices typically contain aggregated interaction frequencies across millions of cells [5], and the dynamic nature of chromatin [6]. These limitations constitute an obvious problem with respect to reconstructing a “consensus” 3D structure.

Several approaches have been proposed to take into account the dynamic nature of chromatin and the aggregated nature of the data. Baù et al. [7] used the Integrative Modelling Platform (IMP) [8, 9] and a Markov Chain Monte Carlo (MCMC) method to simulate a large set of 50,000 independent structural models from 5C data. A subset of the resulting structural ensemble consisting of the 10,000 structures with the best scores was then clustered, such that the different clusters arguably represent the variability of chromatin conformation in the population-averaged data. An MCMC approach for structural ensemble determination from 5C data was also utilized in a study by Rousseau et al. [10], leading to a probabilistic model of the interaction frequency data. This allows for sampling from the posterior distribution of structures after a sufficient number of Monte Carlo steps. IMP has also been used to simulate an ensemble of 10,000 structures, that simultaneously encounter the restraints, assuming that the ensemble represents the dynamic nature of chromatin [3].

Another class of methods for identifying 3D chromatin structure from chromosomal contact data relies on reconstructing a “consensus” 3D structure from a (possibly incomplete and noisy) Euclidean distance matrix (EDM) consisting of pairwise distances (in 3D) between different regions in the genome. In general, this EDM is not known, but is typically estimated from the interaction frequency matrix. Given an EDM various optimization approaches that fall under the general topic of multidimensional scaling (MDS) (see e.g. [11] for an overview) can be used to find an optimal 3D structure. Methods based on MDS are often simpler and can handle larger problems, such as multiple chromosomes or single chromosomes on finer scales, than many of the more complex probability based methods. On the other hand, such methods often ignore the dynamic nature of chromatin and the aggregated nature of the Hi-C data.

The most basic form of MDS is the so-called classical (or metric) MDS, where the optimal coordinate reconstruction from a given EDM is found directly by eigen decomposition of the so-called Gram matrix (see Methods for details). An early application of classical MDS to determine 3D structure from chromosome contact data was presented by Dekker et al. [12]. In general, however, when the EDM has been inferred from interaction frequencies, the MDS approaches consider the reconstruction as a nonlinear and non-convex optimization problem using some iterative optimization method. For example, the EDM has been inferred by assuming simple transformations of genomic distances to Euclidean distances, and an iterative optimization method has been applied to reconstruct the coordinates best corresponding to the EDM [13, 14].

Other optimization methods applied on MDS problems to find coordinates from incomplete distances exploit the rank constraints on the EDM (or corresponding Gram matrix) to find an optimal EDM for the relevant spatial dimension. One successful method in this respect is based on convex semidefinite programming [15, 16], which relaxes the problem to a convex optimization problem. These approaches are applicable to model 3D chromosome configurations [17]; however they cannot handle large problems, due to computational limitations.

Technological improvements have also facilitated the reconstruction of 3D genome structures. In particular, adjustments to the Hi-C protocol have been introduced to enable identification of interactions between chromosome regions in single cells [18]. Single-cell Hi-C, however, inevitably suffers from sparse sampling of chromosomal interactions and a general lack of information on non-local distances between genomic regions with no mutual contacts. Nonetheless, mapped interactions are found in individual cells, potentially enabling a more robust determination of the underlying 3D structure [18].

One way to handle these limitations is to replace missing distances with their ‘shortest-path’ equivalence; that is, considering the existing (observed) entries in the EDM as weighted edges in a graph, and replacing each missing edge weight with the smallest possible sum of weights traversing the graph along the observed edges [19]. One drawback of completing the EDM with the shortest-path distances, however, may be that the imputed distances introduce noise which dominates over the more accurate local distances.

An application of single-cell like contact maps coupled with missing-value imputation using the shortest-path method and classical MDS to find 3D coordinates, was recently proposed [20]. This approach offers an efficient way of establishing 3D genome structures. However, accuracy may be limited both by the noise introduced by the shortest-path procedure as well as from the limitations of the classical MDS approach.

Another approach proven to be effective on many optimization problems relies on optimization on manifolds. The problem of finding optimal coordinates from an EDM can be formulated as an optimization problem on the manifold of the set of positive semidefinite matrices of fixed rank [21, 22]. The Riemannian quotient geometry of the manifold can be exploited to yield efficient algorithms for the optimization problem [23]. However, this strategy has, to our knowledge, not been applied to 3D genome reconstruction in previous studies.

In this paper, we show that the manifold based optimization (MBO) approach can be successfully applied to 3D genome reconstruction. MBO significantly outperforms the simpler methods based on classical MDS in terms of consistency with the original contact map and structural violations, while remaining sufficiently efficient to handle large-scale problems.

Using both simulated and real single-cell Hi-C data, we show that, by combining the shortest-path derived distances with appropriate weights to reduce the influence of noise, MBO can efficiently reconstruct 3D structures consistent with the chromosome contact maps, despite the noisy and sparse nature of the data. Our implementation of the manifold optimization method is based on the Manopt software [24] that provides a Matlab interface for optimization on manifolds.

## Results

In the following sections, we apply MBO to reconstruct the 3D structure of genomes in two types of settings, and compare to two other popular approaches. First, to evaluate the method’s ability to reconstruct a known 3D structure, we consider a given *a priori* 3D structure, and sample contact frequencies from this structure. Then, we apply the methods to recently published single-cell Hi-C data [18], and evaluate the ability of the resulting structural models to reconstruct the original contact maps.

### Manifold based optimization for 3D genome reconstruction

**Fig 1:**
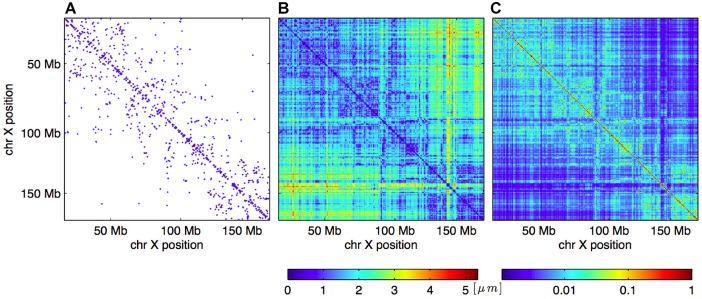
Example of generation of distance and weight matrices for the optimization procedure. A: Original chromosomal contact map (*C*_*ij*_) based on chromosome X from cell 1. A blue dot indicates the presence of an observed interaction in the single-cell Hi-C data set. B: Distance matrix (*D*_*ij*_) consisting of Euclidean distances (in *μ*m) corresponding to the contact map to the left after running the shortest-path algorithm. C: Corresponding weight matrix (*H*_*ij*_), containing numbers between 0 and 1 giving the weight for each of the distances in the Euclidean distance matrix to the left. See the Methods section for details.

Given a matrix of interaction frequencies, typically from a Hi-C or single-cell Hi-C data set, we seek to reconstruct the corresponding 3D coordinates of the genome structure. In classical MDS (CMDS), this reconstruction is performed by converting the contact frequencies into an EDM (Fig 1B), and uses singular value decomposition for direct coordinate reconstruction. Crucially, such approaches assume that all Euclidean distances in the EDM are of equal importance and equally accurate. This is problematic, since it is known that short genomic distances are sampled much more frequently than long genomic distances. Also, in single-cell Hi-C, contacts are restricted to only two interactions per restriction fragment, for autosomal chromosome pairs, resulting in a large number of missing values.

In our method, which relies on manifold based optimization (MBO) [22], the low-rank property of the EDM, and the resulting redundancy in the distances, are exploited to infer the missing distances. We consider the completion of the EDM while simultaneously allowing for missing distances. We do this by combining the shortest-path completed distances with weights, such that imputed (and typically long) distances are weighted less in the subsequent optimization procedure (Fig 1C). This allows for flexibility in the reconstruction of uncertain regions of the final 3D structure, while enforcing distances in more reliable sections of the structure. The Methods section provides an in-depth description of the full algorithm.

### Reconstruction with sparse and noisy distance information

As a first validation of the method, we have considered an *in silico* test case where a known chromosome structure was employed to test the ability of different methods to reconstruct the original structure from incomplete and noisy distance information. Here, MBO is compared to the classical MDS (CMDS) approach recently presented in Lesne et al. [20], where the graph shortest-path method is utilized to replace missing distances. This method is generally known as Isomap [19], while the adaptation to 3D genome reconstruction was named ShRec3D in Lesne et al. [20]. In the following we will refer to this method simply as CMDS. In addition, we present comparison with the ChromSDE method of Zhang et al. [17], which is based on semidefinite programming and is significantly more computationally demanding than both the CMDS method and MBO.

**Fig 2:**
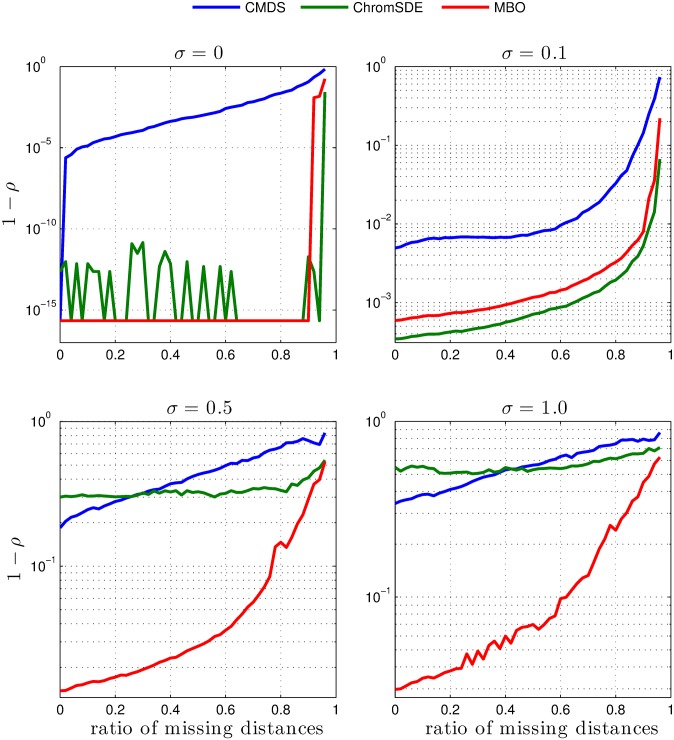
3D genome reconstruction comparisons for the different algorithms. (1–Spearman rank correlation) between the original and reconstructed distances in a single structure of chromosome X from [18], for the different models (CMDS, ChromSDE and MBO) using different noise levels (*σ*) and ratios of missing distances. *σ* = 0 corresponds to the case where no noise was added to the distance matrix, whereas *σ* = 0.1, 0.5 and 1.0, corresponds to cases where increasing levels of Gaussian noise has been added. On the horizontal axis, different levels of missing distances are shown, spanning from 0 (no missing distances) to 0.95 (95% of distances have been removed).

The structure considered in this validation is a 3D model of mouse haploid chromosome X generated from single-cell Hi-C data by Nagano et al. [18]. The 3D model represents chromosome X using a 50 kilo base pair (kbp) resolution. However, for the current test, the structure was re-sampled at 600 kbp, by taking the average spatial position of groups of bins, this due to the computational limitation of the ChromSDE method. Additionally, we evaluate different levels of noise (*σ*), added to the final contact matrix, as well as different levels of contact scarcity (see Methods section). The results from these tests are shown in Fig 2. The data shows the structural similarity between original distances and reconstructed distances for the different methods, for different noise levels (*σ*) and ratios of missing distances.

For the weakly noisy case (Fig 2; *σ* = 0.1) MBO and ChromSDE still reconstruct structures more consistent with the orignal structure than CMDS. For the two cases with higher noise levels, however, MBO performs markedly better, and produces structures more similar to the original, compared to the two other methods (Fig 2; *σ* = 0.5 and 1.0).

In the noiseless case (*σ* = 0) both MBO and ChromSDE are able to reconstruct the original structure exactly as long as a sufficient number of the pair-wise distances are known. This would be expected for ChromSDE, since the semidefinite programming approach is convex in this case. That MBO also recovers the original coordinates exactly is not *a priori* obvious. Naturally, the ratio of distances needed for an exact reconstruction will vary with the size *n* of the problem. In fact, it has been shown that knowledge of *m* ≥ *Cn* ^6/5^ *r* log *n* (for some positive contant *C*) random entries of an *n* × *n* matrix of rank *r* is sufficient for an exact completion of the matrix in most cases [25].

**Fig 3:**
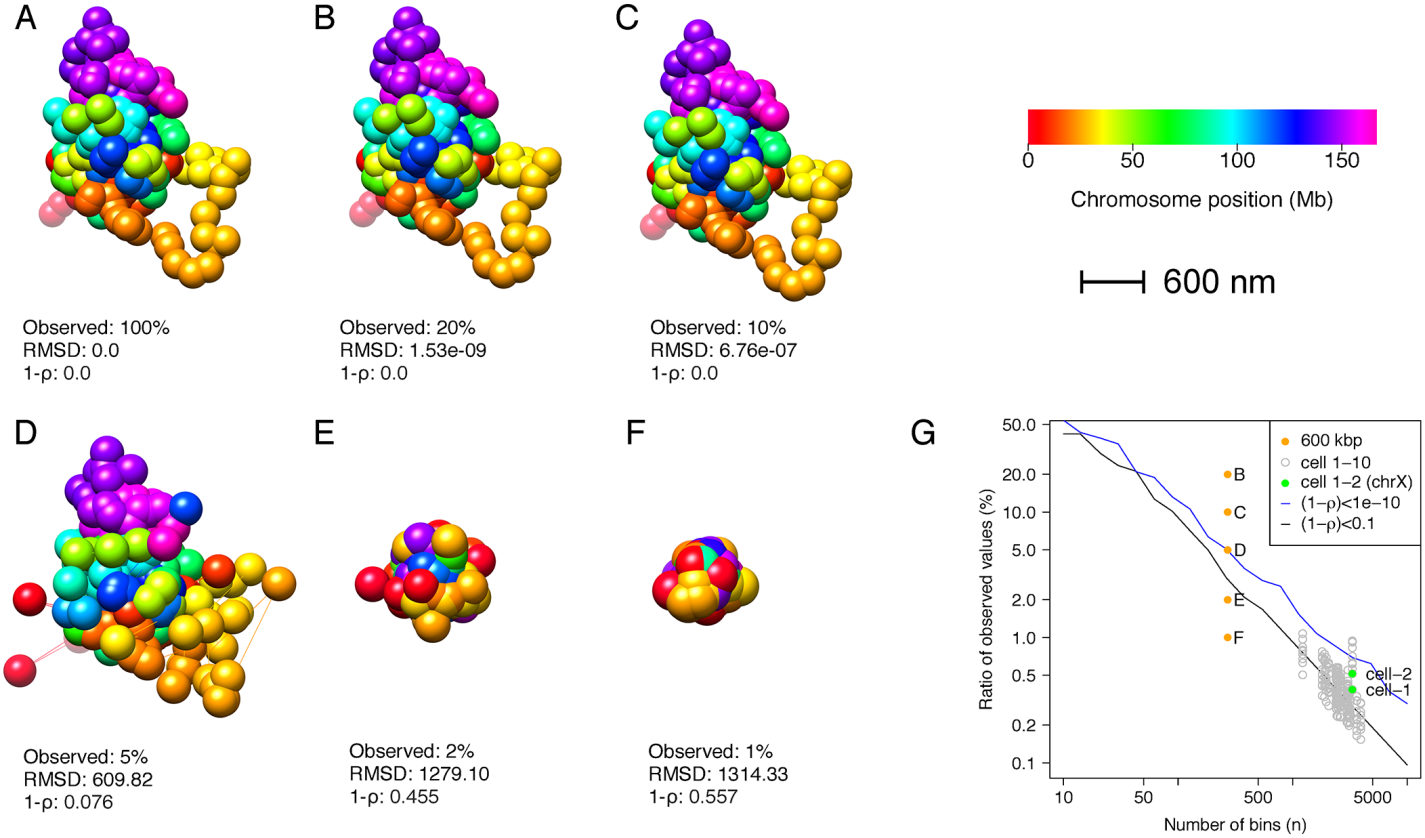
Reconstruction of chromosome X at different levels of observed information. A: Original chromosome X structure from [18], resampled at 600 kbp. B-F: Reconstructed 3D structures of chromosome X, with different ratios of observed distance information (20%, 10%, 5%, 2% and 1%, respectively). Information about the RMSD (in nm) and 1 − *ρ*, compared to the original structure (A) is given below each of the structures in A-F. G: Ratio of observed values as a function of the number of bins *n*, i.e. the size of the structure being reconstructed. The structures in B-F are highlighted (orange dots), and compared to an estimated curve showing the minimum ratio of observed values for complete reconstruction ([1-*ρ*]<1e-10; blue curve) or partial reconstruction ([1-*ρ*]<0.1; black curve). All data from [18] are shown as gray circles, and the X chromosome data sets from cell 1 and cell 2 are highlighted in green.

We inspected the ability of MBO to reconstruct the considered orignal structure when the missing distances approach this limit. The original structure can be exactly reconstructed with up to ∼ 90% missing data (Fig 3A–3C). With 95% missing data, the structure is still similar to the original structure, with an RMSD of ∼ 610 nm. At levels of missing data above 98%, however, the structure collapses into a compact globule, due to missing interactions between distal bins (Fig 3E–3F).

To inspect this dependency further, we calculated the minimum ratio of observed distance values needed for complete reconstruction ([1-*ρ*]<1e-10) and partial reconstruction ([1-*ρ*]<0.1)), for a range of different sampled structures with varying number of bins (*n*) (see Fig 3G). The required percentage of observed interactions is dependent on the total number of bins in the system considered. We compared the structures from Fig 3B–3F with these estimated curves, and indeed found that the compact globular structures correspond to a ratio of observed values crossing the boundary of partial reconstruction. Furthermore, we compared these curves to the sets of all chromosomes from the single-cell Hi-C data from [18]. As can be seen in Fig 3G, the datasets are distributed around the curve of partial reconstruction ([1-*ρ*]<0.1)). This could indicate that the current single cell Hi-C data sets are generally too sparse for high confidence structure reconstruction. Note, however, that the single-cell Hi-C data for chromosome X (cell 1 and cell 2) are between the partial and complete reconstruction curves, and are therefore likely to be among the more reliable data sets for structural reconstruction and method comparisons.

### Computation time

**Fig 4:**
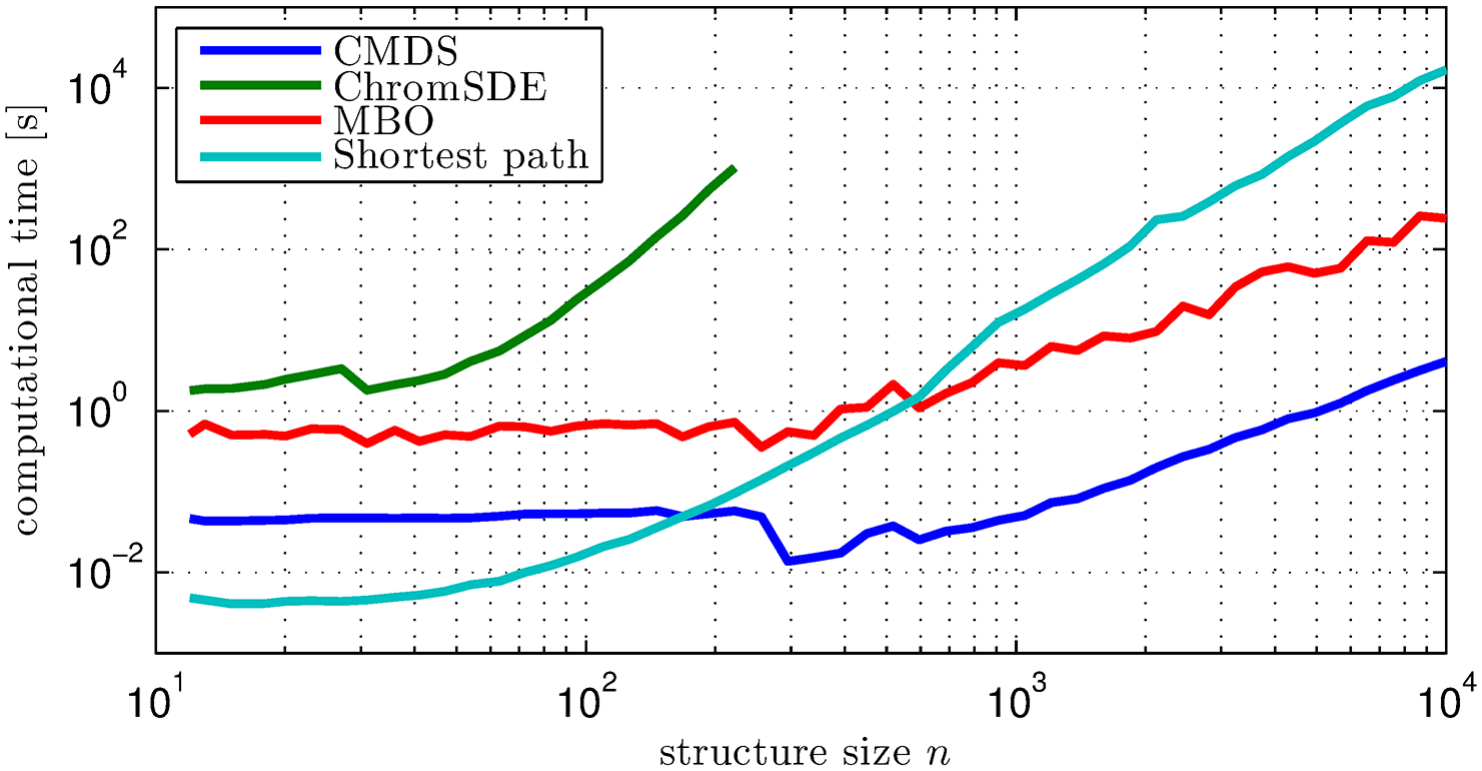
Computational time evaluations for the different algorithms. Computational time (in seconds) for reconstructing a single chromosome structure using three different algorithms CMDS (dark blue), ChromSDE (green), and the MBO algorithm (red) presented here. For comparison, the shortest path algorithm (light blue) is also shown. The computational time is shown as a function of structure size *n*, i.e. the number of bins in the structure.

Typical computation times for the methods considered in the validation performed above are shown in Fig 4, as a function of the problem size *n* (i.e. *n* is the number of bins in the reconstructed structure). As expected, CMDS (excluding the shortest-path algorithm) is fastest, while ChromSDE is slowest. Note, however, that MBO has the same asymptotic behavior as CMDS for large *n*. Further, when the input EDM has missing values, the shortest-path distances must be calculated before application of CMDS. Hence, for *n* larger than about 500, MBO is actually the fastest of the three methods. In practice, using stringent settings, reconstruction of e.g. chromosome X using MBO at 50kbp resolution takes less than 5 minutes.

### Full genome reconstruction reveals a dynamic structure of homologous chromosome pairs

Next, we examined the ability of MBO and CMDS to reconstruct contact maps for the full set of chromosomes, based on single-cell Hi-C data [18]. We therefore applied MBO and CMDS to all mouse chromosomes individually, for two different single cells (named “cell 1” and “cell 2” in [18]), and evaluated the resulting structures. We evaluated and compared the ability of the methods to reconstruct structures with resulting contact maps consistent with the input data, by inspecting the percentage of contacts established in the reconstructed structure that were also present in the original contact map (% correct contacts). Additionally, we evaluated the occurrence of structural inconsistencies in the inferred structures, *i.e*. the percentage of bins being too close to each other (% min distance violation), and the percentage of consecutive bins that are too far away from each other (% connectivity violation). See the Methods section for details.

**Fig 5:**
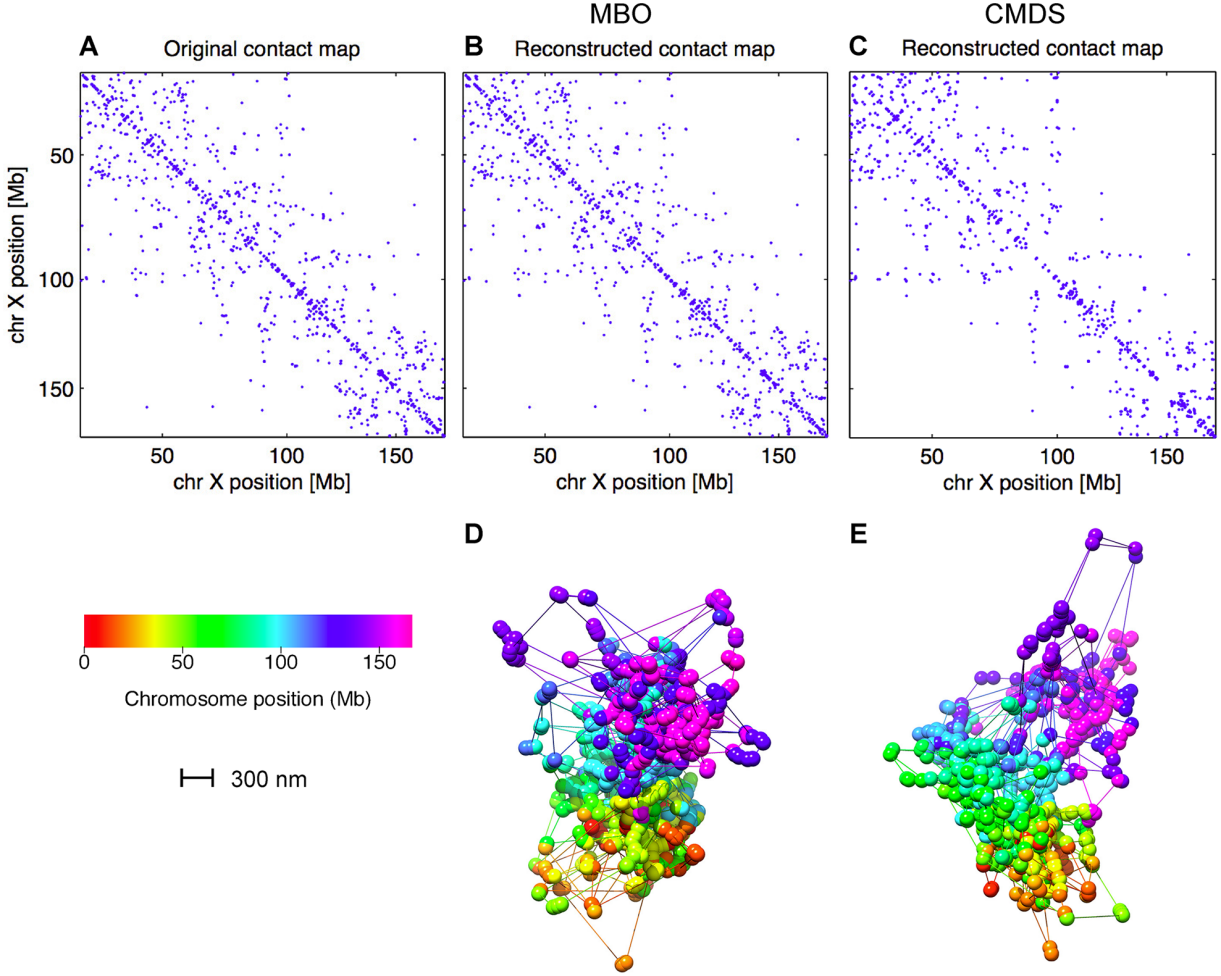
Contact map reconstruction comparison between MBO and CMDS. A: Original contact map. Blue dot indicates the presence of a contact in the single-cell Hi-C data set for chromosome X (cell 1). B: Contact map obtained after 3D reconstruction using MBO, based on the contact map (in A) and then re-calculating the contacts. C: Reconstructed contact map, as in B, but using CMDS. D: Reconstructed 3D structure using MBO, corresponding to the contact map in B. E: Reconstructed 3D structure using CMDS, corresponding to the contact map in C. Each bead in D and E has a diameter of 150 nm. Lines represent connected beads with missing bead position information.

**Fig 6:**
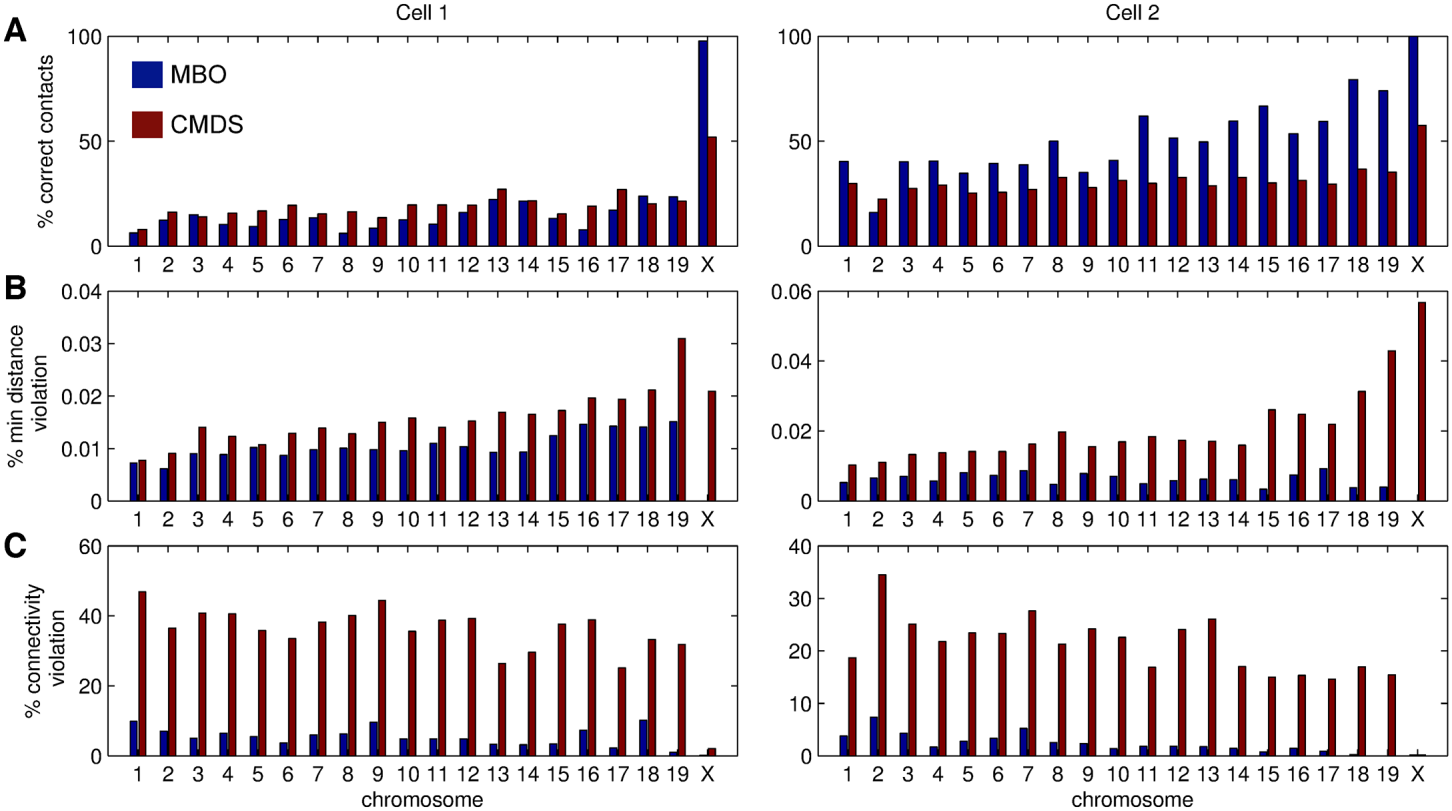
Consistency comparison of reconstructed 3D genome models based on MBO and CMDS. Consistency of the structures obtained from reconstructing all chromosomes for cell 1 (left) and 2 (right) using MBO (blue) and CMDS (red). A: Reconstruction accuracy, given as the percent correct contacts when comparing original and reconstructed contacts maps for different chromosomes. B: Distance violation, given as the occurrence (in percent) of regions in the structures that are below the minimum distance (at 30 nm). C: Connectivity violation, given as the occurrence (in percent) of consecutive regions in the structures that are further away than the maximum distance (200 nm). Blue bars indicate the performance of MBO, while red bars indicate the performance of CMDS.

We started by considering chromosome X, where only one copy is present in the data. For chromosome X, we found that MBO was able to reconstruct the original contact map of the haploid X nearly completely (both cases > 95% reconstructed). CMDS, on the other hand, was not able to reconstruct the contact matrix of chromosome X at more than ∼50–60% correct contacts (Figs 5C and 6A). Similar results were found for all 10 individual cells from [18] (see S1 Fig), even though the percentage of correct contacts was closer to 80% for some of the cells with the fewest number of input contacts (cells 9 and 10).

Interestingly, for homologous chromosome pairs, where two chromosome copies are present, reconstruction was not as consistent with the input contact maps as for chromosome X, as only ∼20% of the contacts in the original maps could be reconstructed (Fig 6A). In other words, the presence of two chromosomal copies affects the ability to reconstruct structures that reflect the original contact matrix. This indicates that the structures of the two homologous copies may contain mutually exclusive contacts, making full reconstruction of the contact maps difficult.

We were interested in investigating the effect of having possibly mutually exclusive contact information from two separate chromosome X structures from cell 1 and cell 2. We therefore randomly sampled 50 new datasets consisting of an equal number of contacts from the matrices from these two cells and inspected the ability of MBO to reconstruct structures corresponding to the resulting contact maps. As S2 Fig shows, the mixed datasets produce structures with a significantly lower percentage of correct contacts, and structures with higher connectivity violations. It should be noted that 3D reconstruction from mixed populations of contact data has no guarantee of reliably estimating a correct structure.

For homologous chromosome pairs, MBO and CMDS performed similarly in terms of percentage of successfully established interactions (Fig 6A). However, when looking at minimum distance violations (chromosomal bins closer than 30 nm, Fig 6B, or violations of the connectivity of consecutive regions (consecutive bins further away than 200 nm, Fig 6C), it is clear that MBO is more successful in positioning the regions in 3D, without imposing obvious violations.

**Fig 7:**
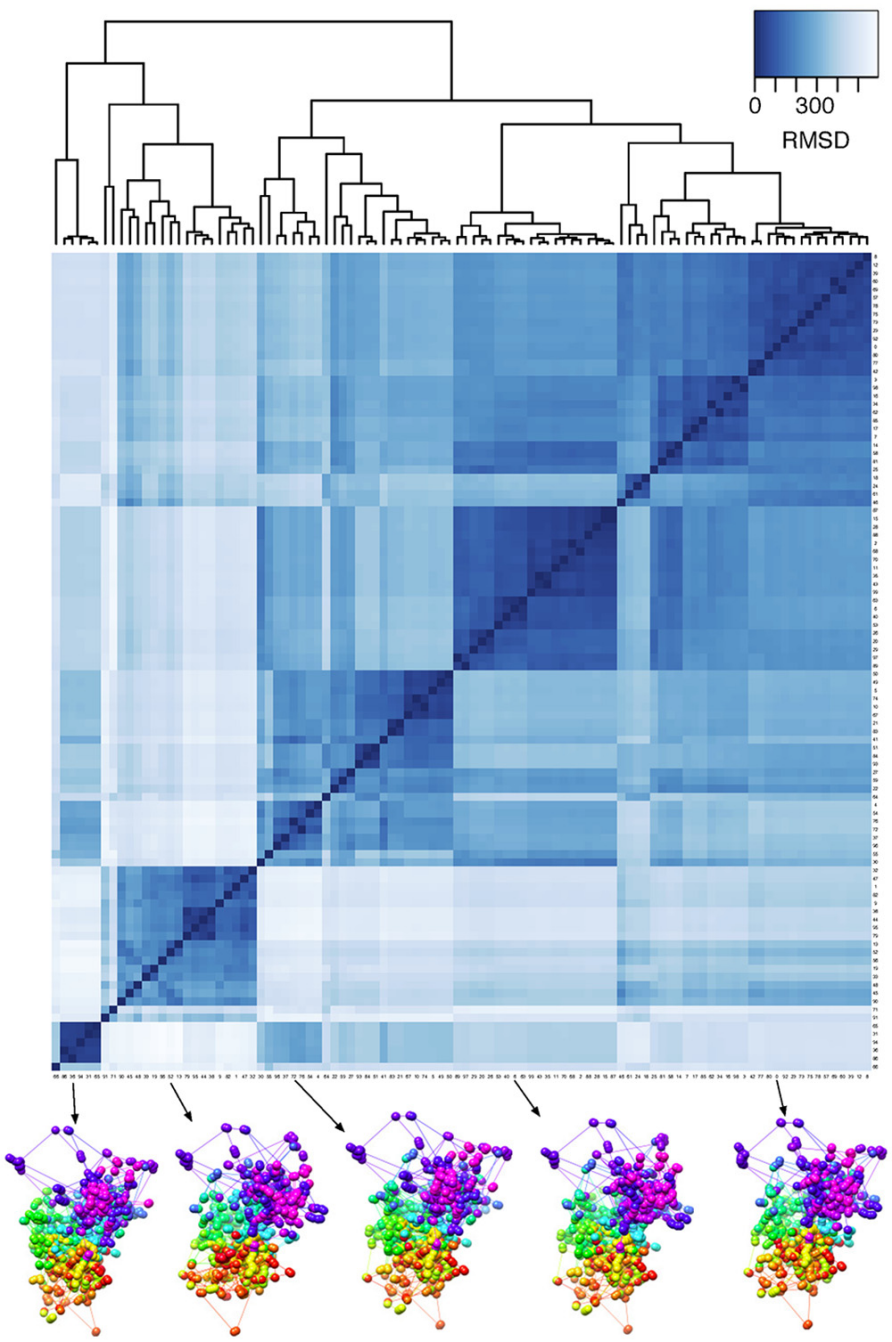
Clustering of chromosome X structural models. The heatmap shows clustered RMSD values between 100 independent optimizations with random initial configurations prior to using MBO on chromosome X. The dendrogram above shows the result of the hierarchical clustering based on the RMSD values. At the bottom, 5 example structures from each cluster are shown.

Since MBO, like most optimization strategies for structural reconstruction, is non-convex, optimized structures might depend on the random starting configuration of the optimization. We wanted to study this effect by running 100 independent optimizations of chromosome X using different random initialization of the starting configurations. We then calculated the root-mean-square deviation (RMSD) between the resulting superimposed structures, and found a high degree of similarity between all the 100 chromosome X structures, with an average RMSD of ∼ 322 nm, similar to what was reported in [18]. Furthermore, we clustered the RMSD values using hierarchical clustering, and the resulting clusters are visualized in Fig 7. As the figure shows, 4–5 large clusters are found, where the structural similarity within the clusters is clearly higher than between clusters, probably reflecting different local optima in the cost function. By inspecting example structures within each of the clusters, overall the similarity between the structures is high. This indicates that the MBO method gives robust results, with similar structures regardless of starting configuration. Nevertheless, it is advisable to run several independent optimizations, to inspect whether the different local optima in the cost function represents disparate structures.

In S3 Fig, the reconstructed 3D structure from chromosome 1 based on MBO is displayed. We note that, despite the presence of two copies, the reconstructed structure shows few structural violations, with minimum distance violation < 0.01% and connectivity violations below 10%. By performing 100 independent reconstructions, as for chromosome X, (see S4 Fig), the average RMSD was found to be ∼ 262 nm. However, for chromosome 1, the resulting clusters were not as clear as for chromosome X, possibly due to the two separate copies of chromosome 1.

For comparison reasons, we applied MBO using a weighting scheme where the shortest-path completed matrix was used directly without accompanying weights. In S5 Fig, the results from this analysis is shown. As the figure shows, using no weights results in a reduced fraction of correct contacts, and additionally, a higher fraction of connectivity violations. The latter point can be explained by considering that all genomic distances are weighted equally when no weights are used. However, when weights are used, as in the MBO method that we present here, short genomic distances will be weighted more, since these will typically contain more contact information. And as a result, connectivity violations will be reduced.

All in all, we have shown that MBO reconstructs 3D structures consistent with the input chromosomal contact data, at the same computational speed as the popular CMDS approach. Additionally, MBO imposes fewer violations relating to the connectivity of the chain, as well as fewer violations from placing regions too close to each other. We have shown that MBO can be used for routine reconstruction of 3D structures from sparsely sampled data, such as single-cell Hi-C.

## Discussion

In contrast to methods such as MCMC and molecular dynamics, methods aiming at reconstructing a single consensus 3D structure can be utilized quickly and in a high-throughput fashion. One challenge with such approaches, however, has been the lack of possibilities for handling the sparse and noisy interaction frequency matrices in a flexible and robust way. In this paper, we have shown that combining weights with manifold based optimization (MBO) allows for reconstructing 3D structures of genomes, even when data are sparse and noisy, such as for single-cell Hi-C. We have shown that the weights allow for prioritization of interactions where information about spatial positioning is found, while allowing the remaining regions to be positioned in a consistent fashion. Specifically, by comparing the reconstructed and original contact maps, we have shown that the single copy of chromosome X in male mouse cells can be reconstructed in a fashion consistent with the input data. For homologous chromosome pairs, however, reconstruction was not complete, most likely due to considerable structural difference between the two chromosome copies.

We note that it is also possible to run MBO on ensemble Hi-C datasets, since the weighing scheme is equally applicable in this case. However, the assumption of a consensus structure would in this case probably be less justifiable, due to the known inherent variability in chromosome interactions across cells in a large population.

As chromosome conformation capture data are becoming increasingly available [26], quick and robust methods for reconstructing chromosomal 3D structure from chromosomal interaction data, are needed. Additionally, for a complete understanding of the mechanisms involved in gene regulation, cell differentiation, DNA replication and repair, genome organization needs to be studied in its correct dimensions. Efficient and robust 3D genome reconstruction tools such as MBO, are likely to play an increasingly important role for such studies in the future.

## Methods

### Theoretical background

A fundamental problem relevant for many applications in various disciplines is to find some coordinates, **x**_*i*_ ∈ ℝ^*r*^, *i* = 1, ⋯, *n* in an *r*-dimensional Euclidean space, given some information about the pair-wise distances between the points. The pairwise distances can be represented by the Euclidean distance matrix (EDM), ***D*** ∈ ℝ^*n*×*n*^, q

$$D_{ij}=\left|\right|x_{i}-x_{j}{\left|\right|}^{2},$$ (1)

which is an *n* × *n* matrix containing the squared distances between the *n* points. By construction the EDM is a symmetric matrix with zero diagonal and non-negative entries satisfying the triangle inequality $\sqrt{D_{ij}}\leq \sqrt{D_{ik}}+\sqrt{D_{kj}}$. Note also that ***D*** is invariant to arbitrary rotations and translations of the set of coordinates **x**_*i*_.

If the EDM is known exactly (without noise or missing entries), the coordinates **x**_*i*_ can be uniquely determined up to arbitrary rotations and translations by introducing the matrix ***B*** ∈ ℝ^*n*×*n*^,

$$B=-\frac{1}{2}\left(I-\frac{1}{n}ee^{T}\right)D\left(I-\frac{1}{n}ee^{T}\right),$$ (2)

where ***I*** ∈ ℝ^*n*×*n*^ is the identity matrix and ***e*** ∈ ℝ^*n*^ is a vector of all ones. If ***D*** is a true EDM in an *r* dimensional space, ***B*** is a symmetric positive semidefinite matrix of rank *r*. That is, ***B*** has maximum *r* nonzero eigenvalues, and ***B*** = ***V*** **Λ** ***V*** ^*T*^, where **Λ** ∈ ℝ^*r*×*r*^ is the diagonal matrix with the *r* nonzero eigenvalues of ***B*** on the diagonal, and ***V*** ∈ ℝ^*n*×*r*^ is the matrix with the *r* eigenvectors of ***B*** as its columns. It can then be shown that $X=V\sqrt{\Lambda }$ is an *n* × *r* matrix with the coordinates **x**_*i*_ as its rows. It is easy to see that ***B*** = ***XX*** ^*T*^, thus ***B*** contains the inner product of the coordinates and is often called the Gram matrix for the set of coordinate vectors.

In many practical applications, however, the EDM may contain noisy and missing values. In this case, finding optimal coordinates **x**_*i*_ must be treated as an optimization problem of finding coordinates that minimize some cost function based the known distances. If all pair-wise distances between points are known, but not necessarily accurately, one solution to the optimization problem is given in terms of classical multidimensional scaling (CMDS). CMDS basically solves the optimization problem of finding a matrix $\hat{B}$ that solves

$$\min_{\hat{B}\in {𝓢}_{+}^{n}\left(r\right)}\left|\right|\hat{B}-B{\left|\right|}^{2},$$ (3)

where ${𝓢}_{+}^{n}(r)$ is the set of positive semidefinite *n* × *n* matrices of rank *r* or less, and ***B*** is the matrix derived from the EDM by using Eq (2). This problem has a closed-form solution in terms of the *r* largest eigenvalues and corresponding eigenvectors of ***B***, namely $\hat{B}=V\Lambda V^{T}$, where **Λ** is now the diagonal matrix with the *r* largest eigenvalues of ***B*** on the diagonal, and ***V*** is the matrix with the corresponding eigenvectors of ***B*** as its columns. Consequently, the corresponding coordinates are given by $\hat{X}=V\sqrt{\Lambda }$. Obviously, if ***D*** is a true EDM for the relevant dimension *r*, there will be exactly *r* nonzero eigenvalues and the procedure reduces to the one described in the previous paragraph, and the coordinates are recovered exactly up to arbitrary rotations and translations. However, if ***D*** is not close to a true EDM, CMDS is often not robust since the nearest distances are measured through ***B*** rather than on ***D*** directly.

### Formulation of the manifold based optimization (MBO) approach

A manifold based optimization approach for the completion of Euclidean distance matrices was recently presented in Mishra et al. [22]. They solved a minimization problem in the form

$$\min_{\hat{D}\in {𝓔}^{n}\left(r\right)}\frac{1}{2}||H⊙\left(\hat{D}-D\right){\left|\right|}^{2},$$ (4)

where 𝓔^*n*^(*r*) is the set of EDMs with embedding dimension *r* or less, ***H*** is a symmetric weight matrix with binary entries (i.e. a matrix whose elements are either 0 or 1) and where ⊙ denotes the element-wise (Hadamard) product between matrices.

For the application of this approach to the case of the 3D genome reconstruction we have applied a slightly more general framework where the weights are allowed to take any non-negative values (not restricted to 0 and 1). In addition, we choose to minimize the differences between the ordinary Euclidean distances rather than the squared distances used in Eq (4). This choice is motivated by the fact that the longer genomic distances will be weighted less in the final optimization, and results in improved performance compared to using squared distances (see S6 and S7 Figs). Thus, we consider the minimization problem

$$\min_{\hat{D}\in {𝓔}^{n}\left(r\right)}\frac{1}{2}||H⊙\left(\sqrt{\hat{D}}-\sqrt{D}\right){\left|\right|}^{2},$$ (5)

where square roots here and in the following denote the element-wise square root of the matrix. Following Mishra et al. [22], Eq (5) can alternatively be formulated as an optimization problem on the set of positive semidefinite matrices with fixed rank, denoted ${𝓢}_{+}^{n}(r)$, by using the mapping from the set ${𝓢}_{+}^{n}(r)$ to the set of EDMs 𝓔^*n*^(*r*) given by

$$D=\kappa \left(B\right)=be^{T}+eb^{T}-2B,$$ (6)

where ***b*** is the vector with the diagonal entries of ***B***, i.e ***b*** = diag(***B***) = (***B*** ⊙ ***I***)***e***. As discussed above a positive semidefinite matrix of rank *r* admits the factorization ***B*** = ***XX*** ^*T*^, where ***X*** ∈ ℝ^*n*×*r*^ and *rank*(***X***) = *r*. Thus, the cost function that we wish to minimize may be written

$$f\left(X\right)=\frac{1}{2}||H⊙\left(\sqrt{\kappa (XX^{T})}-\sqrt{D}\right){\left|\right|}^{2}.$$ (7)

Note that the ***X*** that minimizes Eq (7) is in fact the coordinates that we wish to find.

To minimize Eq (7) we have implemented a solver for the optimization problem in Matlab using the Manopt toolbox [24] using the symfixedrankYYfactory(n, r) manifold, which provides the geometry for the manifold of *n* × *n* positive semidefinite matrices with rank *r*.

Manopt includes a number of different solvers for the optimization problem. Here we will employ a trust-region solver which, unlike steepest descent, utilizes information about both the gradient and the Hessian of the cost function, and has been shown to have good convergence rates. The gradient of *f*(***X***) can be written

$$\mathrm{grad}f\left(X\right)=\kappa^{*}\left(H^{\left(2\right)}⊙\left(ee^{T}-K\right)\right)X,$$ (8)

where ***H*** ^(2)^ = ***H*** ⊙ ***H*** is the matrix with the squared weights and the matrix ***K*** is the symmetric matrix with zero diagonal and off-diagonal entries given by

$$K_{ij}=\sqrt{\frac{D_{ij}}{\kappa {\left(XX^{T}\right)}_{ij}}},\;i\neq j.$$ (9)

κ*(***B***) is the adjoint operator of *κ* defined by

$$\kappa^{*}\left(B\right)=2\left(\mathrm{Diag}\left(Be\right)-B\right),$$ (10)

where *Diag*(***v***) = (***vee*** ^*T*^) ⊙ ***I*** is the function that returns the *n* × *n* matrix with the *n* × 1 vector ***v*** on the diagonal and zeros elsewhere.

In addition to the gradient the trust-region algorithm also requires the Hessian in a given direction ***U***, Hess *f*(***X***)[***U***]. One can show that the Euclidean Hessian of *f*(***X***) takes the form

$$\mathrm{Hess}f\left(X\right)\left[U\right]=\kappa^{*}\left(H^{\left(2\right)}⊙\left(ee^{T}-K\right)\right)U+\frac{1}{2}\kappa^{*}\left(H^{\left(2\right)}⊙G⊙\kappa \left(XU^{T}+UX^{T}\right)\right)X,$$ (11)

where ***G*** is the symmetric matrix with zero diagonal and off-diagonal entries

$$G_{ij}=\sqrt{\frac{D_{ij}}{{\left(\kappa {\left(XX^{T}\right)}_{ij}\right)}^{3}}},\;i\neq j.$$ (12)

The conversion from the Euclidean to the Riemannian Hessian, needed for the optimization algorithm, is performed internally in Manopt. For additional details about the manifold based algorithm, see [22, 24].

### Reconstructing a known 3D structure

From the known 3D structure. a true EDM was constructed containing the pair-wise squared distances between all the 600 kbp sized bins. To model the uncertainty and possible sparsity of distance information inferred from chromosomal contact data such as Hi-C, the original distance matrix was contaminated by adding random noise as well as randomly removing a given percentage of the distances. That is, from the original Euclidean distance matrix ***D*** (containing the squared pair-wise distances), a noisy and incomplete set of distances *δ*_*ij*_ is generated as

$$\delta_{ij}=\delta_{ji}=\sqrt{D_{ij}}\left|1+\sigma \epsilon_{ij}\right|,\;\text{for}\;(i,j)\in 𝓝$$ (13)

where *ϵ*_*ij*_ are sampled randomly from a standard normal distribution and where 𝓝 is the set of entries (*i*, *j*) for which the distances are available.

Tests were run for different values for the noise level *σ* and ratio of missing distances (size of 𝓝).

### Inferring 3D structure from single cell Hi-C data

The raw results from a single-cell Hi-C experiment typically lists a number of observed contacts between specific genome positions. From the raw results, the contacts were aggregated into equally spaced bins along the chromosomes. For the results presented here a bin size of 50 kbp was used. Then all observed contacts were assigned to their corresponding bins. In the case that multiple contacts fell into the same bin, the duplicate entries were ignored so that a binary contact matrix *C*_*ij*_ was obtained for each chromosome. Hence, *C*_*ij*_ = 1 represents a Hi-C contact between bins *i* and *j*, while *C*_*ij*_ = 0 represents the absence of a contact.

To use the MBO approach, the binary contact map must be converted into a distance matrix *D*_*ij*_. First a target distance *d*_*c*_ is assigned to all bins with an observed Hi-C contact. Secondly, the connectivity along the chromosome is taken into account by assigning a distance *d*_*n*_ to neighboring bins. Hence, as a first step the following matrix is constructed

$$D_{ij}=\{\begin{array}{cc} d_{c} & \;\text{if}\;C_{ij}=1,\\ d_{n} & \;\text{if}\;C_{ij}=0\;\text{and}\;\left|i-j\right|=1,\\ 0 & \;\text{elsewhere}\;. \end{array}$$ (14)

Since the MBO method works also for incomplete distance matrices, the optimization could in principle be run directly on Eq (14), letting the weights *H*_*ij*_ be nonzero only for the nonzero entries of *D*_*ij*_. However, since only the local distances (contacts and neighboring bins) are known, a direct optimization of Eq (14) would lead to a very compact structure where all bins are located close together. Hence, for a consistent 3D structure some information about the large distances must be included. One possible method is to assign large distances and small weights to the non-interacting bins (see e.g. [27, 28]). The large distances will then act as a repulsive force and counteract the formation of a compact state. Another possibility is to apply the shortest-path method to fill the missing entries of the distance matrix. In this way the missing distances may take more realistic values since they are deduced directly from the known distances. However, these shortest path-distances still introduce noise that may seriously influence the result. Motivated by the fact that the shortest-path derived distances are more noisy than the ‘original’ contact-distances that we wish to satisfy, we have adopted a slightly more flexible approach where we combine the shortest-path completed matrix with weights so that the shortest-path inferred distances are weighted less in the optimization procedure.

Thus, we first replace the zero entries in *D*_*ij*_ with the shortest-path derived distances. We then introduce the weight matrix *H*_*ij*_ whose elements are chosen to be inverse proportional to the number of edges traversed in the shortest path, i.e $H_{ij}=n_{ij}^{-q}$ where *n*_*ij*_ is the number of edges that is needed to connect node *i* and *j*. That is, the original distances will have weights equal to one, while the shortest-path derived distances will have smaller weights. The value *q* is a factor that specifies the relative magnitude of the weights for the non-observed edges compared to the observed ones, and was found by maximizing the percent correct contacts and minimizing distances violations (see S8A Fig for an example). In our case this value was always found to be between 1 and 3 (see S1 File), but in theory, for other data, the optimal value may be outside this range. Here, we have used a simple optimization scheme by trying out a range of values for *q*. This is likely sufficient in most cases, since the effect of using different values for *q* on the final structures is not very large. For example, on chromosome X for cell 1, using a range of values of q between 0 and 3, the structures all had RMSD<300nm compared to the structure with optimized *q* (see S8B Fig).

MBO is initialized by starting with a random initial configuration (a random point on the manifold), and convergence is considered obtained if the cost function or the norm of the gradient drops below a small value (1e-20 and 1e-08, respectively). After a successful convergence of the optimization algorithm the resulting coordinates *x*_*i*_ are scaled to best agree with the original contact map. That is, we search for a scaling constant *c*_*l*_ so that ${\hat{D}}_{ij}=||c_{l}x_{i}-c_{l}x_{j}||$ contains exactly *n*_*c*_ pair-wise distances smaller than the contact distance *d*_*c*_, where *n*_*c*_ is the number of contacts in the original contact matrix. Note that in the case of perfect agreement, the contact matrix derived from the coordinates *c*_*l*_ *x*_*i*_ will be identical to the original contact matrix, since the number of entries are the same. The optimal value for *c*_*l*_ is found by a simple binary search method.

The percent correct contacts was calculated by direct comparisons of original and reconstructed contact matrices. Minimum distance violations were defined as the percent fraction of pairwise distance below 30 nanometers. Connectivity violations were defined as the percent fraction of neighboring (connected) bins with a distance above 200 nanometers. In Eq 14, *d*_*c*_ = 60nm, *d*_*n*_ = 120nm.

### Implementation

MBO is implemented in Matlab, and is based on the Manopt software [24]. Code is freely available at http://folk.uio.no/jonaspau/mbo/.

## Supporting Information

S1 Fig Consistency comparison of reconstructed 3D genome models based on MBO and CMDS for ten single cells. Consistency of the structures obtained from reconstructing all chromosomes for cell 1–10 using MBO (blue) and CMDS (red). Top panel: Reconstruction accuracy, given as the percent correct contacts when comparing original and reconstructed contacts maps for different chromosomes. Middle panel: Distance violation, given as the occurrence (in percent) of regions in the structures that are below the minimum distance (at 30 nm). Bottom panel: Connectivity violation, given as the occurrence (in percent) of consecutive regions in the structures that are further away than the maximum distance (200 nm). Blue bars indicate the performance of MBO, while red bars indicate the performance of CMDS.
(PDF)

S2 Fig Consistency of reconstructed chromosome X 3D models, based on MBO, using data from a mixed population of cell 1 and cell 2. Left panel: Reconstruction accuracy, given as the percent correct contacts when comparing original and reconstructed contacts maps. Right panel: Connectivity violation, given as the occurrence (in percent) of consecutive regions in the structures that are further away than the maximum distance (200 nm). Red dots corresponds to a 3D reconstruction of chromosome X from cell 1, and blue dots corresponds to a 3D reconstruction of chromosome X from cell 2. The purple circles correspond to optimizations from 50 independent randomly sampled data sets with equal amounts of contacts from cell 1 and cell 2. The thick purple line indicates the median, while the thin purple lines indicates the 25th and 75th percentiles.
(PDF)

S3 Fig Structural model of chromosome 1. A: Reconstructed 3D structure using MBO, where each bin is represented as a bead with a diameter of 150 nm. B: Same reconstructed 3D structure as in A, but where each bin is connected by a line to show the trace of the chromosomal structure. C: Original contact map. Blue dot indicates the presence of a contact in the single-cell Hi-C data set for chromosome 1 (cell 1). D: Contact map obtained after 3D reconstruction using MBO and then re-calculating the contacts.
(PDF)

S4 Fig Clustering of chromosome 1 structural models. The heatmap shows clustered RMSD values between 100 independent optimizations with random initial configurations prior to using MBO on chromosome 1. The dendrogram above shows the result of the hierarchical clustering based on the RMSD values. At the bottom, 5 example structures are shown.
(PDF)

S5 Fig Consistency comparison of reconstructed 3D genome models based on MBO and CMDS, when no weights are used during the optimization. Consistency of the structures obtained from reconstructing all chromosomes for cell 1 (left) and 2 (right) using MBO without weights (blue) and CMDS (red). Top panels: Reconstruction accuracy, given as the percent correct contacts when comparing original and reconstructed contact maps for different chromosomes. Middel panels: Distance violation, given as the occurrence (in percent) of regions in the structures that are below the minimum distance (at 30 nm). Bottom panels: Connectivity violation, given as the occurrence (in percent) of consecutive regions in the structures that are further away than the maximum distance (200 nm).
(PDF)

S6 Fig Consistency comparison of reconstructed 3D genome models based on MBO and MBO-squared. Consistency of the structures obtained from reconstructing all chromosomes for cell 1 using MBO (blue) and MBO with squared distances in Eq (4) (MBO-squared; red). A: Reconstruction accuracy, given as the percent correct contacts when comparing original and reconstructed contact maps for different chromosomes. B: Distance violation, given as the occurrence (in percent) of regions in the structures that are below the minimum distance (at 30 nm). C: Connectivity violation, given as the occurrence (in percent) of consecutive regions in the structures that are further away than the maximum distance (200 nm). Blue bars indicate the performance of MBO, while red bars indicate the performance of CMDS. Panels D-F show the same statistics as for A-C, respectively, but when no weights are used during the optimization (essentially setting *q* = 0 in the weight matrix).
(PDF)

S7 Fig 3D genome reconstruction comparisons for MBO with squared distance terms. Same as Fig 2, but also showing the performance of MBO using squared distances in Eq (4) (MBO-squared, in cyan).
(PDF)

S8 Fig Example of procedure to find the optimal value for *q*. A: To find the optimal *q* for a given reconstruction with MBO, we try out a range of values (*e.g*. 0–3). The optimal *q* is given by the maximum value of the (% correct contacts)-(% min distance violation) (red circle). B: RMSD values (nm) for the same structures as in A, compared to the optimal structure (red circle).
(PDF)

S1 File Tab-delimited file containing optimized values of *q* used for final 3D reconstruction of chromosomes from cell 1. Column 1: chromosome, column 2: optimized *q* for the MBO method, column 3: optimized *q* for the MBO method using squared distances in Eq (4) (MBO-squared).
(TXT)
