# Supplementary figures and images for: Manifold Based Optimization for Single-Cell 3D Genome Reconstruction
Source: PLoS Comput Biol. 2015 Aug 11;11(8):e1004396. doi: 10.1371/journal.pcbi.1004396 (PMC4532452; doi:10.1371/journal.pcbi.1004396)

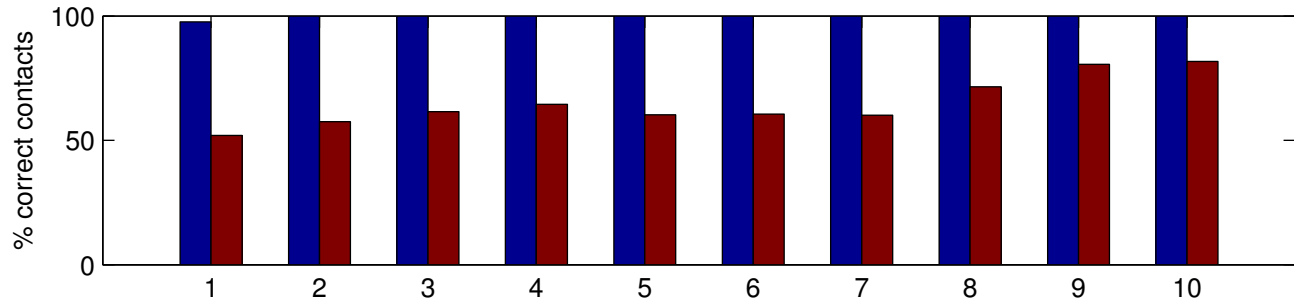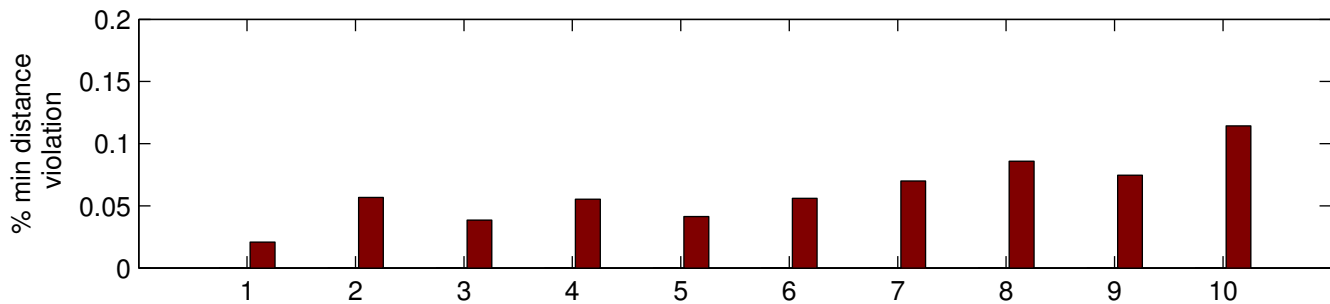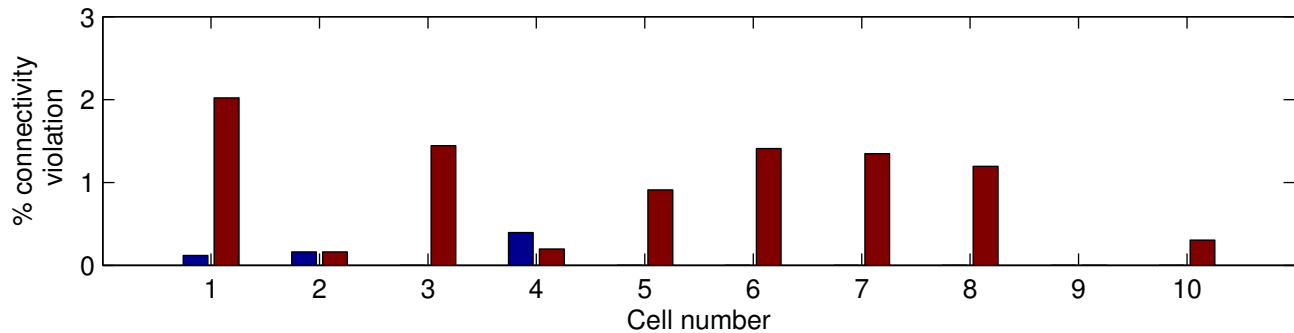

Supplement: S1 Fig — Consistency of the structures obtained from reconstructing all chromosomes for cell 1–10 using MBO (blue) and CMDS (red). Top panel: Reconstruction accuracy, given as the percent correct contacts when comparing original and reconstructed contacts maps for different chromosomes. Middle panel: Distance violation, given as the occurrence (in percent) of regions in the structures that are below the minimum distance (at 30 nm). Bottom panel: Connectivity violation, given as the occurrence (in percent) of consecutive regions in the structures that are further away than the maximum distance (200 nm). Blue bars indicate the performance of MBO, while red bars indicate the performance of CMDS. (PDF) [file pcbi.1004396.s001.pdf]

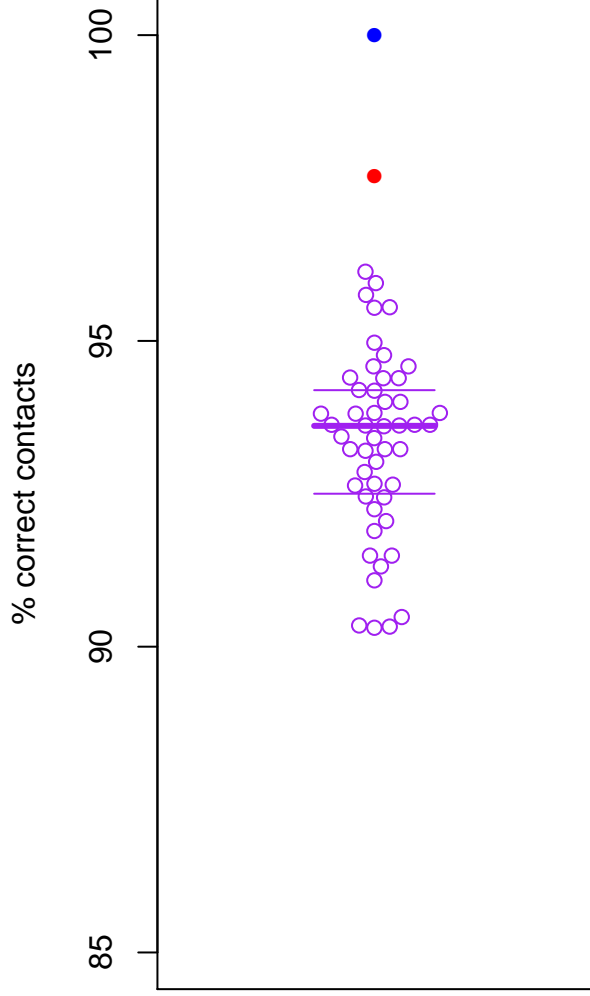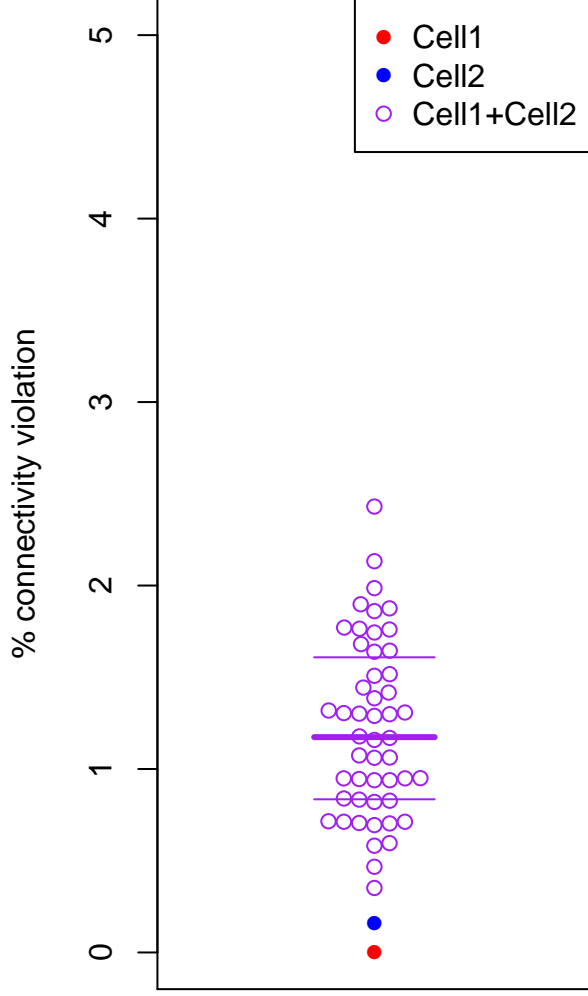

Supplement: S2 Fig — Left panel: Reconstruction accuracy, given as the percent correct contacts when comparing original and reconstructed contacts maps. Right panel: Connectivity violation, given as the occurrence (in percent) of consecutive regions in the structures that are further away than the maximum distance (200 nm). Red dots corresponds to a 3D reconstruction of chromosome X from cell 1, and blue dots corresponds to a 3D reconstruction of chromosome X from cell 2. The purple circles correspond to optimizations from 50 independent randomly sampled data sets with equal amounts of contacts from cell 1 and cell 2. The thick purple line indicates the median, while the thin purple lines indicates the 25th and 75th percentiles. (PDF) [file pcbi.1004396.s002.pdf]

**A**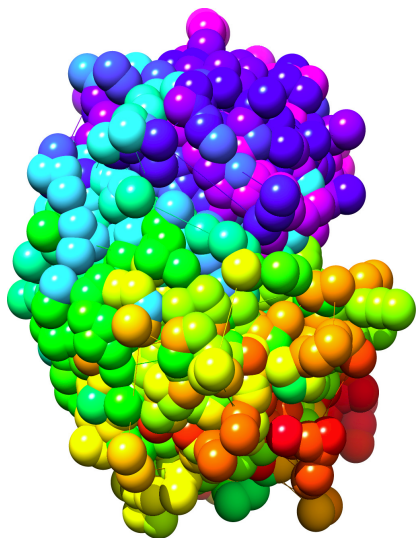

— 300 nm

**B**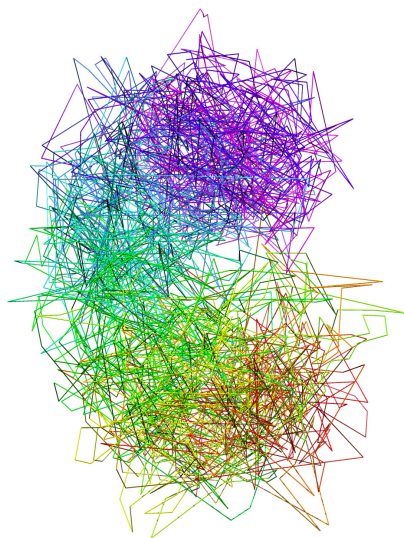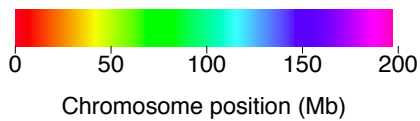**C**

Original contact map

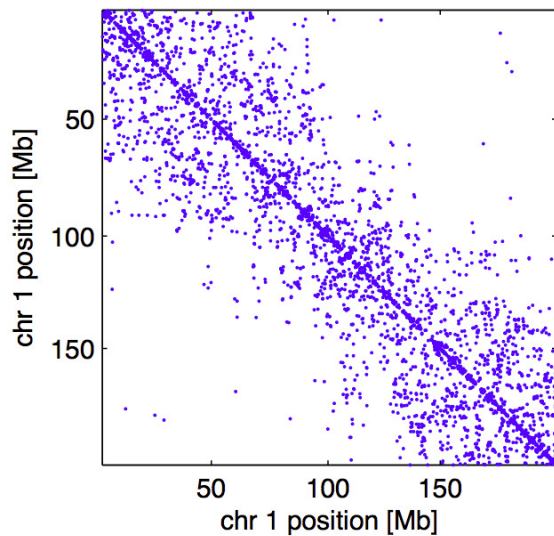**D**

Reconstructed contact map

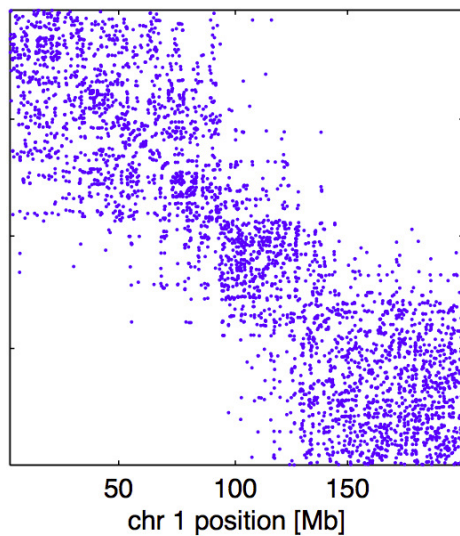

Supplement: S3 Fig — A: Reconstructed 3D structure using MBO, where each bin is represented as a bead with a diameter of 150 nm. B: Same reconstructed 3D structure as in A, but where each bin is connected by a line to show the trace of the chromosomal structure. C: Original contact map. Blue dot indicates the presence of a contact in the single-cell Hi-C data set for chromosome 1 (cell 1). D: Contact map obtained after 3D reconstruction using MBO and then re-calculating the contacts. (PDF) [file pcbi.1004396.s003.pdf]

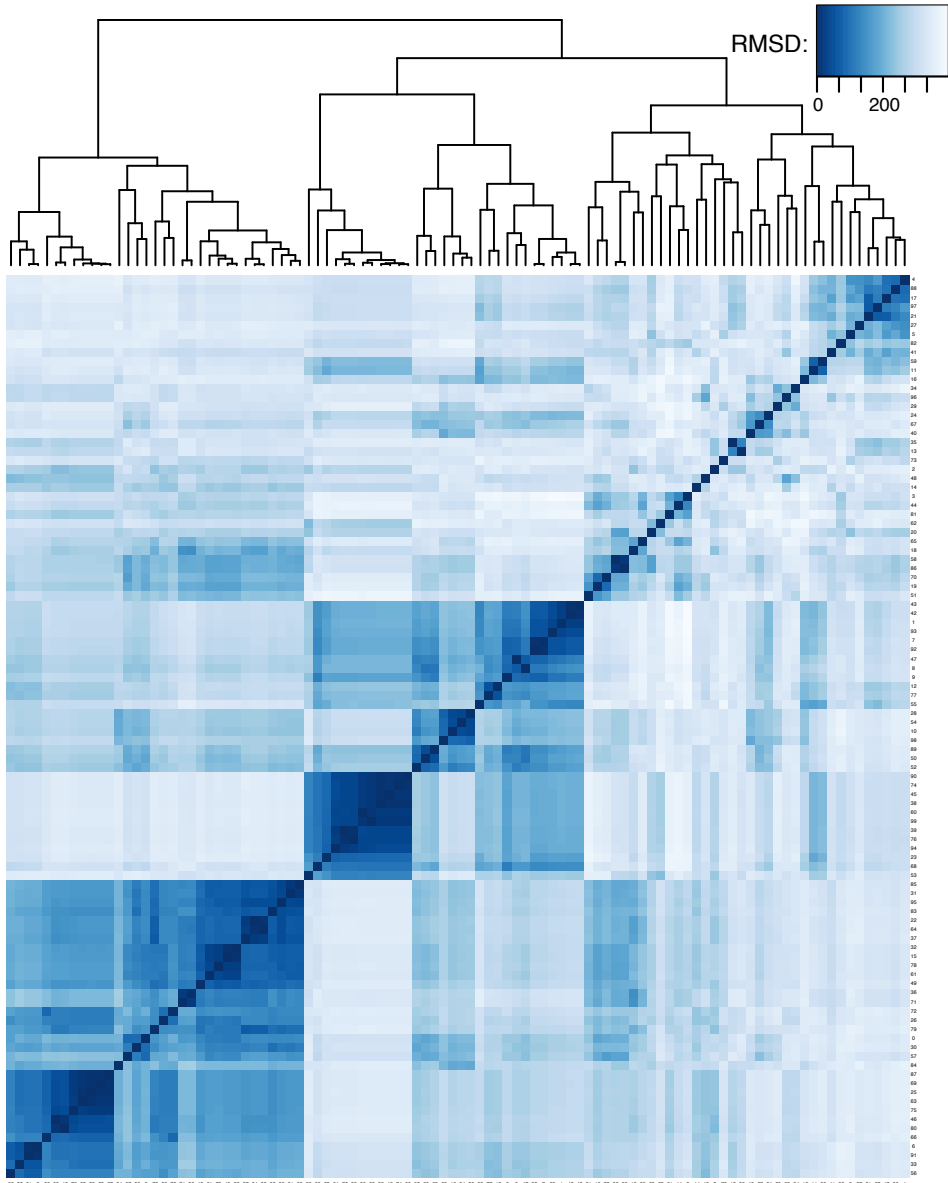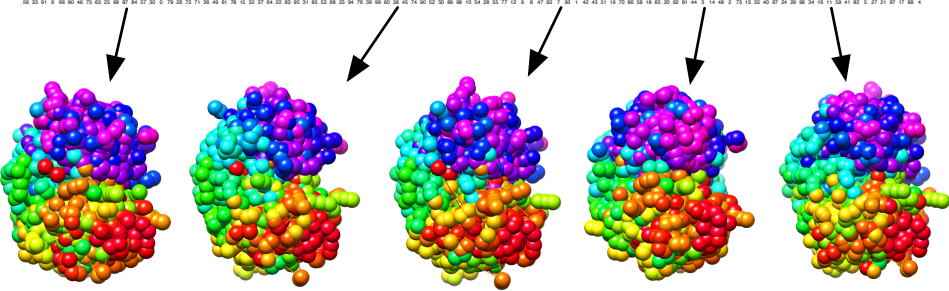

Supplement: S4 Fig — The heatmap shows clustered RMSD values between 100 independent optimizations with random initial configurations prior to using MBO on chromosome 1. The dendrogram above shows the result of the hierarchical clustering based on the RMSD values. At the bottom, 5 example structures are shown. (PDF) [file pcbi.1004396.s004.pdf]

Cell 1

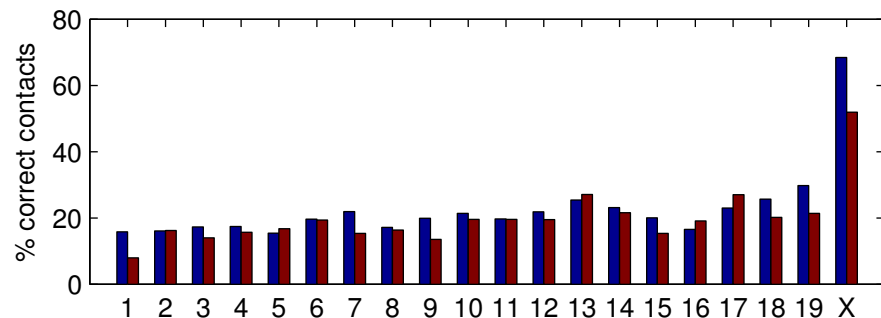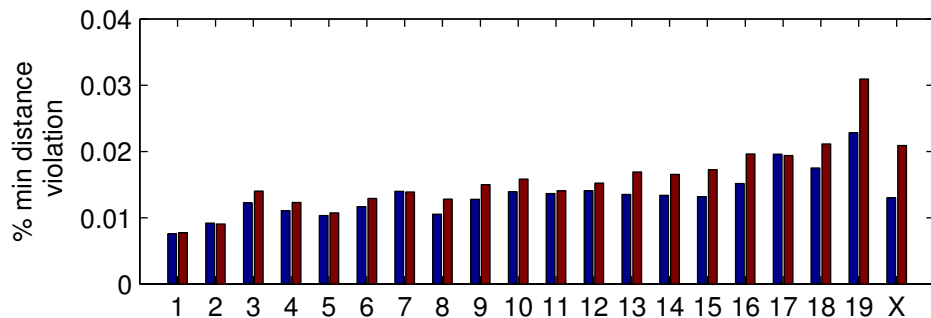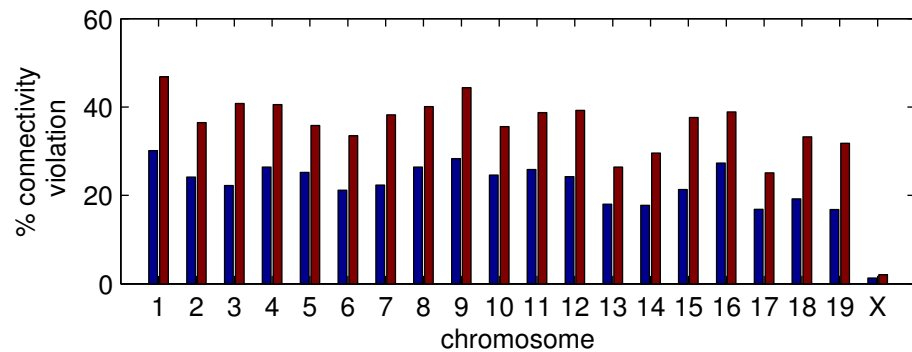

Cell 2

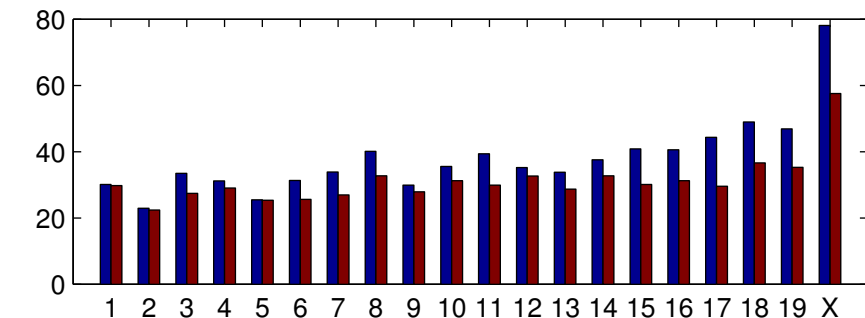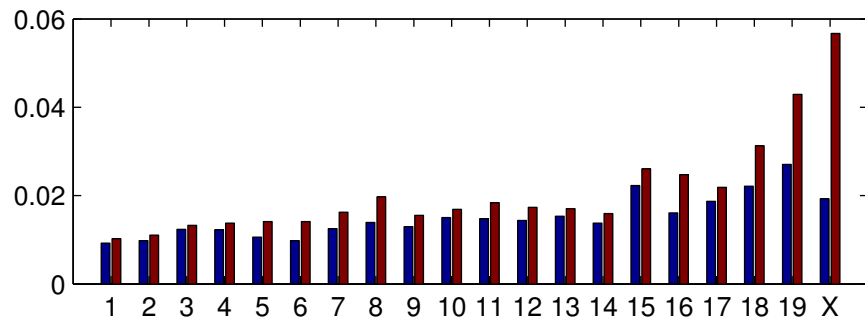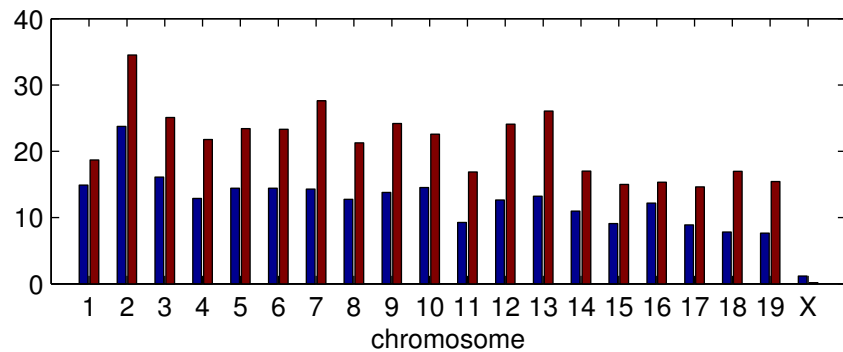

Supplement: S5 Fig — Consistency of the structures obtained from reconstructing all chromosomes for cell 1 (left) and 2 (right) using MBO without weights (blue) and CMDS (red). Top panels: Reconstruction accuracy, given as the percent correct contacts when comparing original and reconstructed contact maps for different chromosomes. Middel panels: Distance violation, given as the occurrence (in percent) of regions in the structures that are below the minimum distance (at 30 nm). Bottom panels: Connectivity violation, given as the occurrence (in percent) of consecutive regions in the structures that are further away than the maximum distance (200 nm). (PDF) [file pcbi.1004396.s005.pdf]

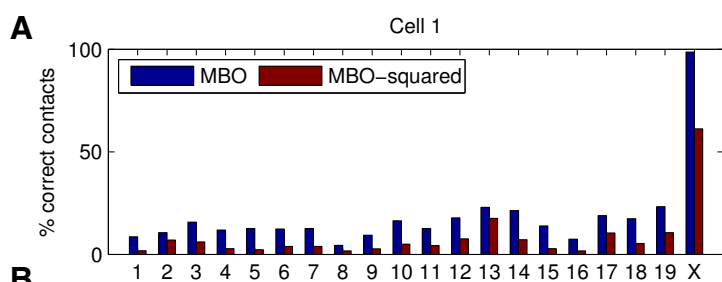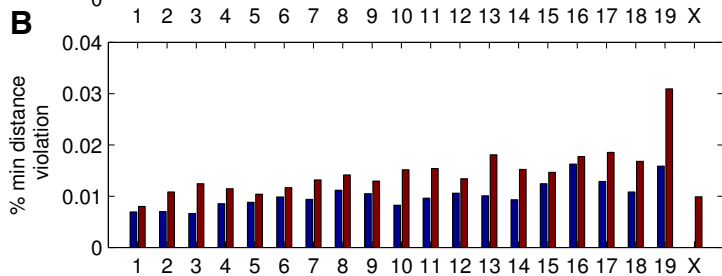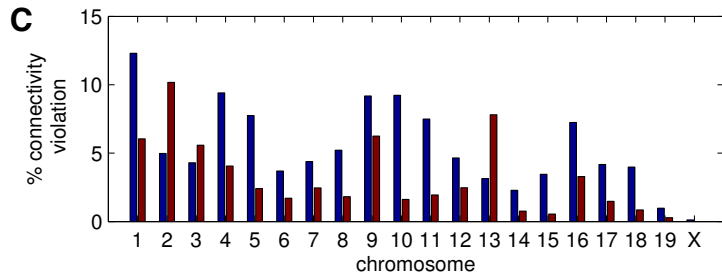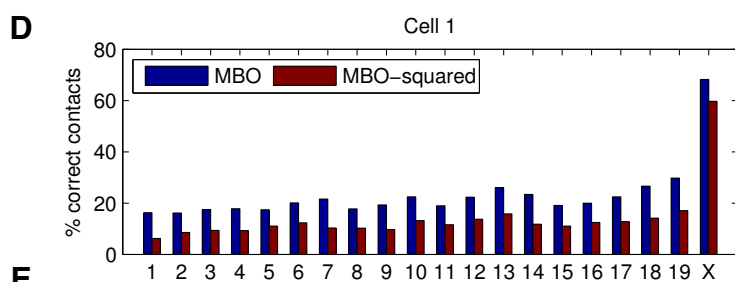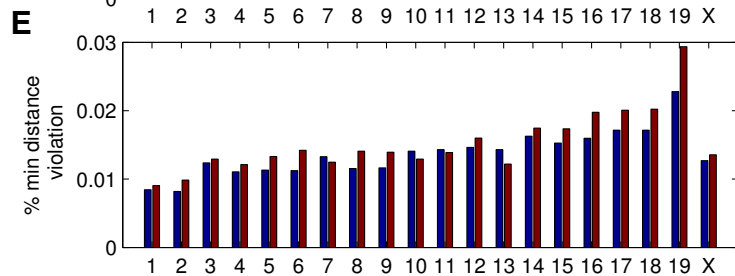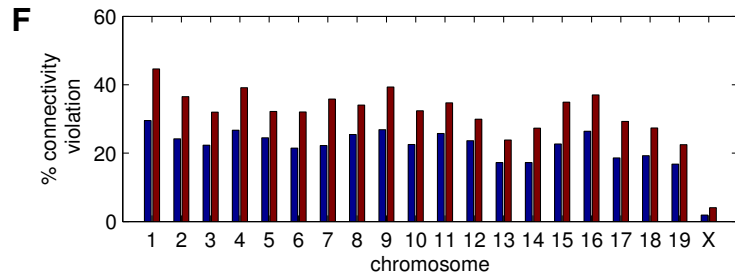

Supplement: S6 Fig — Consistency of the structures obtained from reconstructing all chromosomes for cell 1 using MBO (blue) and MBO with squared distances in Eq (4) (MBO-squared; red). A: Reconstruction accuracy, given as the percent correct contacts when comparing original and reconstructed contact maps for different chromosomes. B: Distance violation, given as the occurrence (in percent) of regions in the structures that are below the minimum distance (at 30 nm). C: Connectivity violation, given as the occurrence (in percent) of consecutive regions in the structures that are further away than the maximum distance (200 nm). Blue bars indicate the performance of MBO, while red bars indicate the performance of CMDS. Panels D-F show the same statistics as for A-C, respectively, but when no weights are used during the optimization (essentially setting q = 0 in the weight matrix). (PDF) [file pcbi.1004396.s006.pdf]

— CMDS — ChromSDE — MBO — MBO-squared

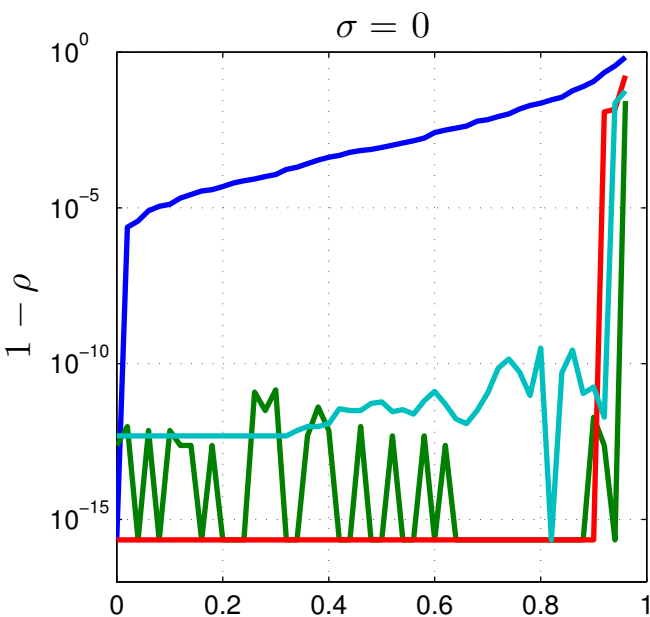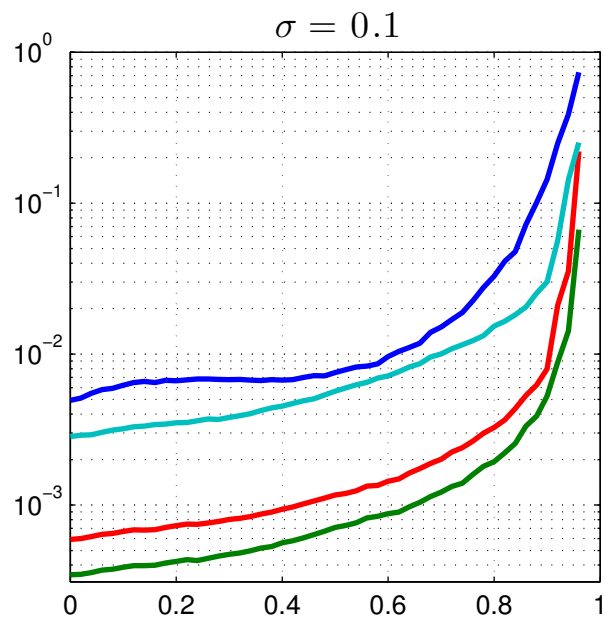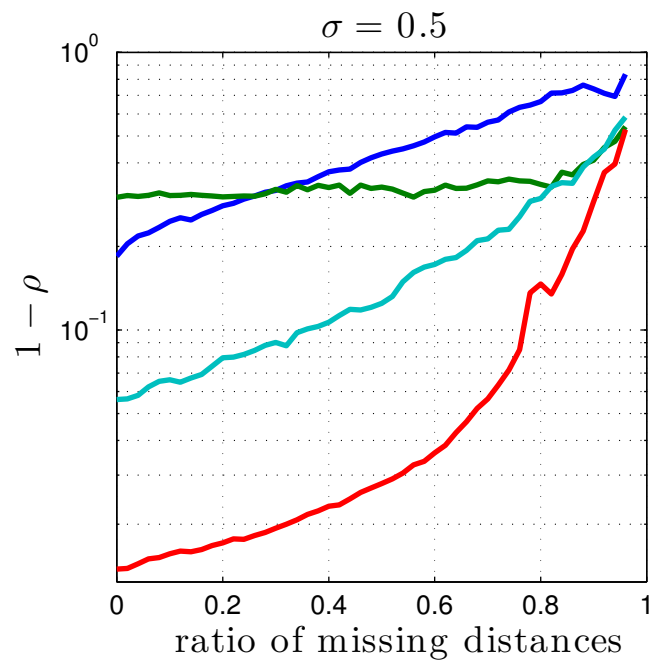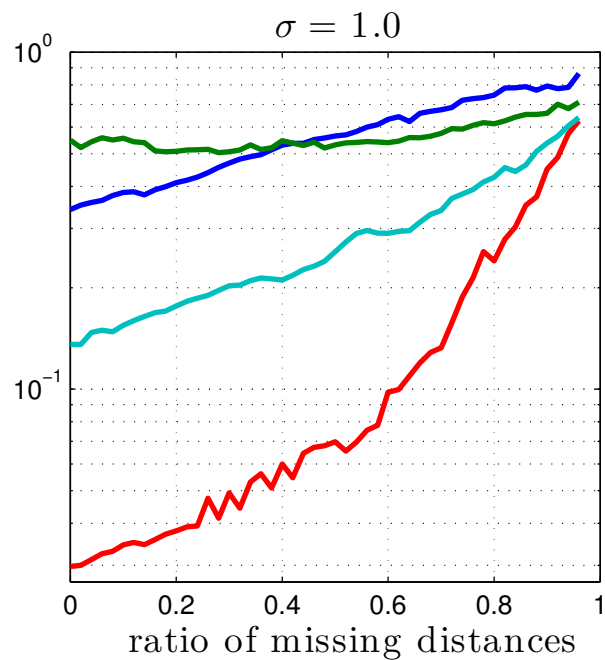

Supplement: S7 Fig — Same as Fig 2, but also showing the performance of MBO using squared distances in Eq (4) (MBO-squared, in cyan). (PDF) [file pcbi.1004396.s007.pdf]

A

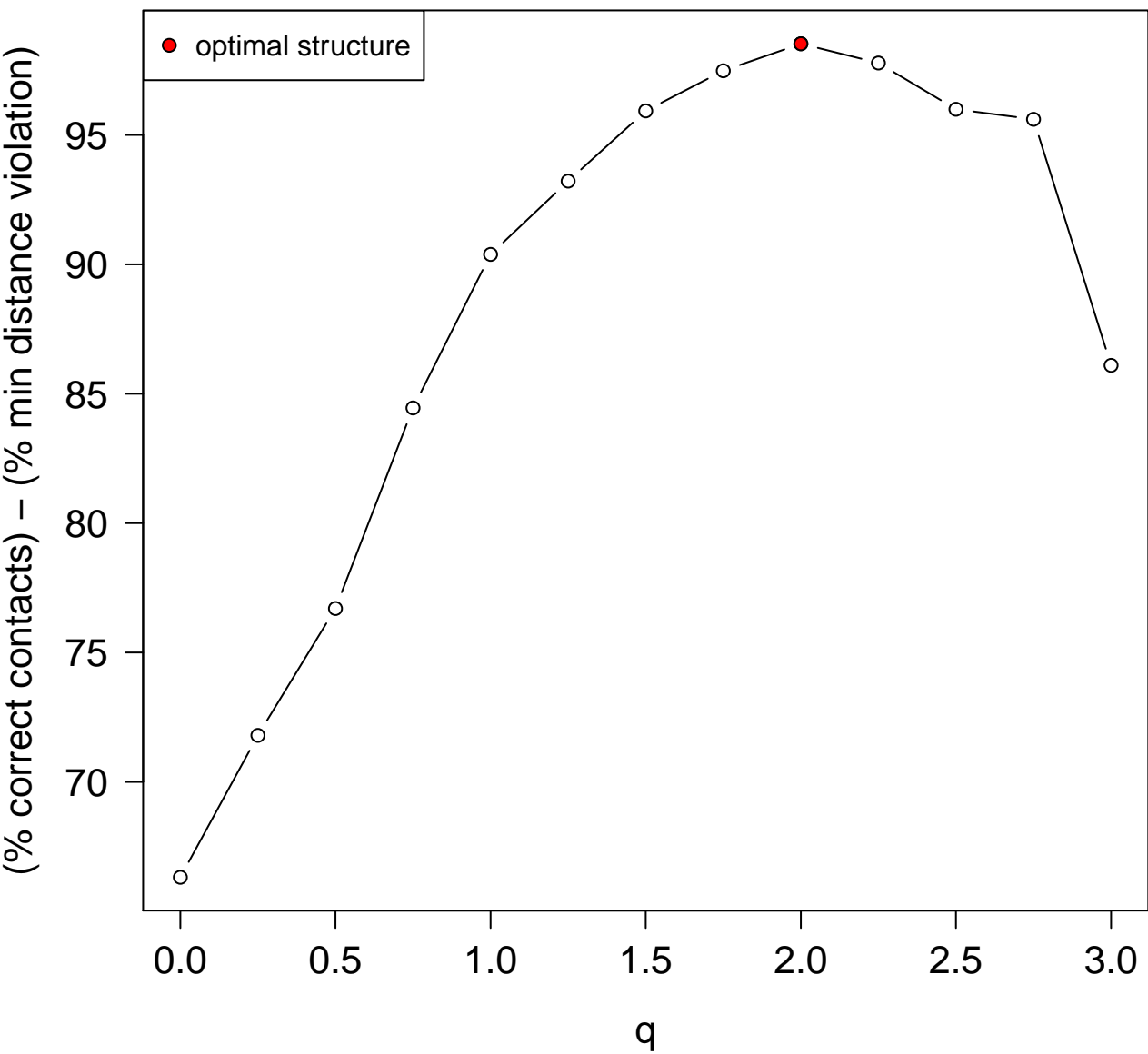

B

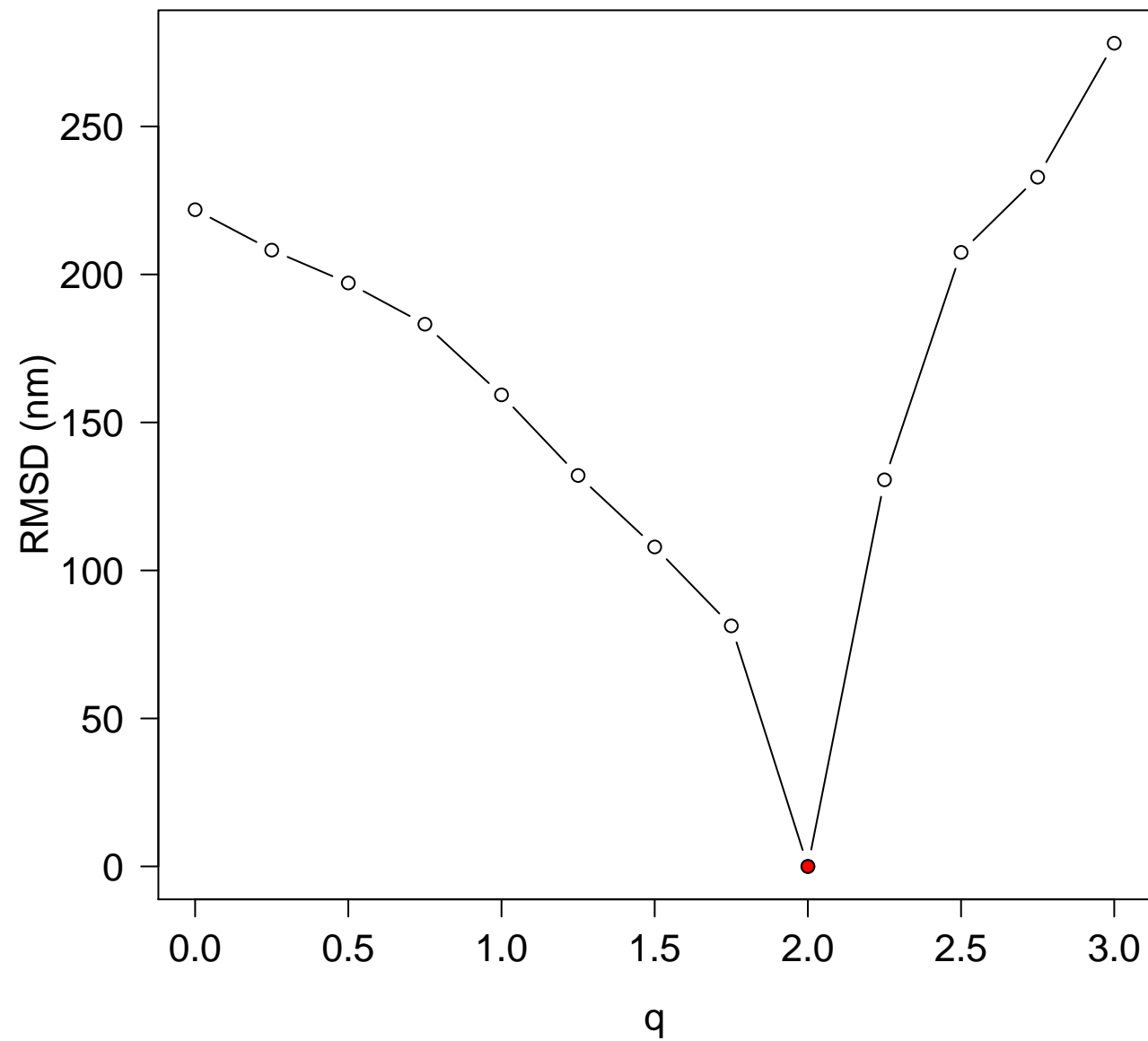

Supplement: S8 Fig — A: To find the optimal q for a given reconstruction with MBO, we try out a range of values (e.g. 0–3). The optimal q is given by the maximum value of the (% correct contacts)-(% min distance violation) (red circle). B: RMSD values (nm) for the same structures as in A, compared to the optimal structure (red circle). (PDF) [file pcbi.1004396.s008.pdf]
